# AMPK Activators as a Drug for Diabetes, Cancer and Cardiovascular Disease

**DOI:** 10.4172/2167-7689.1000118

**Published:** 2014-04-03

**Authors:** Mark Goodman, Zhenling Liu, Ping Zhu, Ji Li

**Affiliations:** 1 Department of Pharmacology and Toxicology, School of Medicine and Biomedical Sciences, State University of New York at Buffalo, Buffalo, NY 14214, USA; 2 Department of Cardiovascular Surgery, Guangdong Cardiovascular Institute, Guangdong General Hospital, Guangdong Academy of Medical Sciences, Guangzhou 510080, China

## Abstract

The cellular mechanisms of AMP-Activated Protein Kinase (AMPK) activators in the treatment and prevention of diabetes, cancer, and cardiovascular disease

## Graphical Abstract

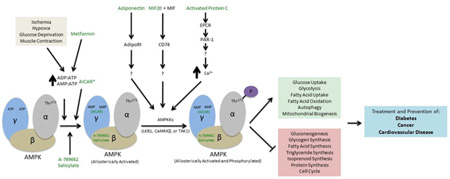

## Introduction

AMP-Activated Protein Kinase (AMPK) is a protein kinase modulating energy metabolism. It was first identified in 1973 for its regulative effect in acetyl-CoA carboxylase (ACC) and 3-hydroxy-3-methyl-glutaryl (HMG)-CoA reductase [1,2]. The name of AMPK was adopted in 1989 to indicate its positive regulation by AMP nucleotide [3]. Since the discovery of AMPK, intense interest has been drawn exponentially to this major fuel gauge as a modulator of the cellular response to energy dysfunction. Agonists of AMPK such as aspirin, metformin, adiponectin, MIF (macrophage migration inhibitory factor), Activated Protein C etc. have been used to investigate the signal pathway. Such a pathway may potentially explain the strong association between cellular energy supply and various diseases such as diabetes, cancer, and cardiovascular disease. This review aims to discuss the mechanisms of AMPK agonists in diabetes, cancer, and cardiovascular diseases, therefore to shed light on the pros and cons of currently available AMPK activators.

## Structure of AMPK

AMPK is a widely distributed and highly conserved heterotrimetric complex composed of a catalytic α (63KDa) subunit and the non-catalytic β and γ subunits which are responsible for the regulation of the kinase activity, enzyme stability, and localization [4]. The N-terminal half of the α catalytic subunit is highly conserved among species and contains a serine/threonine kinase domain followed by an Auto-Inhibitory Sequence (AIS), a C-terminus containing the subunit interacting domains required for binding to β subunit (β-SID), and a conserved leptomycin-sensitive Nuclear Export Sequence (NES) [5].

The β subunit consists of a domain identified as Glycogen-Binding Domain (GBD). While the function of the GBD is still not clear it may serve to co-localize AMPK with downstream targets such as glycogen synthase [6]. The second domain of β subunit is called ASC or SBS (Subunit Binding Sequence), which interacts with both α and γ subunits [7].

The γ subunit includes the domains of allosteric regulation by AMP/ADP/ATP. It is composed of two pairs of motifs called CBS because of their relationship to cystathioine-β-synthase sequences. Recent studies have clarified the mode of nucleotide binding to CBS in yeast and mammalian cells [8,9]. AMP and ATP play the role of allosteric activator and inhibitor respectively through binding to CBS1 and CBS3.

## Upstream Activators of AMPK

Current research suggests that there are at least three upstream AMPK kinases (AMPKKs) in mammals. The first one is LKB1 which includes STRAD α (STE20-related adaptor protein α) and MO25 (scaffolding mouse 25 protein). It is the most widely expressed and indispensable for the gluconeogenic flux and glucose homeostasis [10,11]. Another AMPKK is Ca2+-calmodulin-protein kinase β (CaMKK β) which can phosphorylate and activate AMPK in response to increased intracellular Ca2+ concentrations, independent of any change in cellular AMP/ATP ratio [12]. The third AMPKK is transforming growth factor-β-activated kinase1 (TAK-1) which regulates AMPK activity by phosphorylating Thr172. Genetic studies in yeast show that TAK1 may directly phosphorylate AMPK [13], but the intrinsic mechanism remains elusive.

## Downstream Targets of AMPK

AMPK regulates energy homeostasis both through direct phosphorylation of metabolic proteins and through signaling changes in gene expression. Some of the catabolic, energy-producing pathways AMPK upregulates include glucose uptake, glycolysis, fatty acid uptake, fatty acid oxidation, and autophagy [14]. AMPK also upregulates mitochondrial biogenesis; this aids the cell in energy production via some of the pathways just mentioned. Complementary to the upregulation of many catabolic pathways, AMPK also inhibits anabolic, energy-consuming pathways including gluconeogenesis, glycogen synthesis, fatty acid synthesis, triglyceride synthesis, isoprenoid synthesis, ribosomal RNA synthesis, and DNA replication associated with the cell cycle [14]. In addition to its wide ranges of effects on energy homeostasis in the cell, AMPK also functions directly as an important energy sensor for cells. AMPK is activated in response to metabolic stress on the cell that lower the energy state of the cell by either inhibiting ATP production (i.e. ischemia, hypoxia, glucose deprivation) or accelerating ATP consumption (i.e. muscle contraction) [15]. Specifically, an increase in ADP: ATP and AMP: ATP ratios activate AMPK [16]. Increases in these ratios cause ADP and AMP to bind to the γ subunit of AMPK, displacing ATP. Conformational changes occur which both promote phosphorylation and inhibit dephosphorylation of AMPK [16]. AMP binding additionally causes an allosteric activation of AMPK [16]. Thr172 at the N-terminal of the α subunit is the site at which AMPK is phosphorylated, activating AMPK [17].

## Drug Mechanisms of AMPK Activation

Many kinds of AMPK activators have been identified and studied. Drugs that target AMPK can activate AMPK either directly or indirectly. Direct activation of AMPK entails binding of the molecule to AMPK causing allosteric activation, promoting phosphorylation of Thr172, and/or inhibiting dephosphorylation of Thr 172. Indirect activation occurs from the molecule increasing the AMP: ATP and ADP: ATP ratios in the cell, often through inhibiting mitochondrial ATP production, and as a result, AMPK is activated by AMP and ADP as described earlier. Additionally, other activators have been identified to activate AMPK through unique pathways that are not as well characterized. Considering the extensive effects of AMPK within the cell it is no surprise that there are many and varied activators of AMPK. Many of these activators show promise for the treatment of a wide variety of health issues including diabetes, cancer, and cardiovascular disease. A selection of these activators are described in more detail below that show the highlight the diversity of activators in both origin, structure, and mechanism.

## Metformin

Metformin is the most commonly used drug for the treatment of type 2 diabetes [18]. In intact cells, metformin up-regulates AMPK activity, and thus increases fatty acid oxidation and down-regulates lipogenic genes, decreases hepatic glucose production and stimulates glucose uptake [19]. The related mechanism has been hypothesized that metformin activates AMPK by inhibiting complex I of the respiratory chain, resulting in a fall in cellular ATP concentration and an increase in the AMP: ATP ratio [20], therefore inhibiting dephosphorylation of AMPK and potentiating the phosphorylation of AMPK by the upstream kinase LKB1. Therefore, AMPK is deemed to be the core mediator of the glucose-lowering effect of metformin. AMPK may act as a potential therapeutic target in the prevention and treatment of type 2 diabetes and insulin resistance.

Recently metformin gained more popularity for the retrospective data suggesting that metformin was associated with a 30% lower cancer incidence [21]. Insulin resistance, obesity and type 2 diabetes are all related to an increased risk of cancer, resulting in higher mortality. Through activating AMPK, metformin can inhibit anabolic process including mTORC1 dependent protein biosynthesis, which in turn activates catabolic process and reduces energy consuming process including cell proliferation [22]. Judging from research of p53 wild-type colorectal cancer cells, metformin-induced inactivation of mTOR by AMPK can also activate autophagy [23]. Study of endometrial, ovary, breast, pancreatic, lung, prostate and colon cancer, acute myeloid leukemia and glioma also showed the potential anti-tumorigenic effect of metformin [23–31]. There are also studies indicating that metformin can relieve heart ischemia and reperfusion injury, independent from its glucose-lowering effect, and its cardio-protective effect is mediated by activation of the Reperfusion Injury Salvage Kinase (RISK) pathway, activation of AMPK and by an increased formation of adenosine. In addition, metformin can modulate several cardiovascular risk factors and reduces the development of heart failure in murine models. Consequently, treatment with metformin might potentially improve cardiovascular outcome in patients at risk for myocardial ischemia, even if these patients do not have diabetes [32].

## Adiponectin

Adiponectin is a 30-KDa secretory protein secreted by adipocytes and was identified in the mid-1900s [33]. Circulating adiponectin has a wide range of multitimers, including trimers, hexamers, and High Molecular Weight (HMW) multitimers [34,35]. Adipo R1 and Adipo R2 serve as receptors of adiponectin. Studies of specific deletions in Adipo R1 or Adipo R2 demonstrate that Adipo R1 primarily mediates stimulation of AMPK phosphorylation, and Adipo R2 mediates PPAR alpha activity [36,37].

Adiponectin improves insulin sensitivity and reduces the adverse effects of inflammatory mediators in vascular cells, and the High Molecular Weight (HMW) multitimers may contain the highest potentcy [38,39]. Adiponectin-KO mice have higher incidence to insulin resistance with high-fat feeding, and treatment with adiponectin can improve insulin sensitivity [40]. Direct correlation between adiponectin levels and proteinuria has been reported, however, there is conflicting data about adiponectin levels and mortality in patients with CKD or coronary artery disease [41–44]. Adiponectin protects against albuminuria through an Adipo R1 receptor pathway by stimulating AMPK and inhibiting ROS (Reactive Oxygen Species), but its relationship with Adipo R2 is still unknown [45]. Recent studies have suggested that adiponectin has an effect in maintaining normal podocyte structure, and relieving cardiovascular injury [46–50]. Adiponectin deficiency can exacerbate the transition from cardiac hypertrophy to heart failure during pressure overload because of disruption of AMPK-dependent angiogenic regulatory axis [51]. Adiponectin regulates the expression of the tumor suppressor gene LKB1 and that LKB1 is required for AMPK activation in human and mouse colon cancer cell lines [52].

## Activated Protein C

Activated Protein C (APC) is a vitamin-K dependent serine protease that down-regulates the blood clotting pathway by cleaving factors Va and VIIIa, which are required for thrombin and thrombomodulin in the blood clotting process [53,54]. In addition to its anticoagulant function, APC also reveals potent cytoprotective and anti-inflammatory action by decreasing expression of pro-inflammatory cytokines and inhibiting interaction and migration of leukocytes across the endothelium by inhibiting the NF-κB cascade [55,56]. APC is a safe topical agent for healing chronic lower leg ulcers in patients with diabetes [57]. APC binds to Endothelial Protein C Receptor (EPCR), and subsequently activates Protease-Activator Receptor 1(PAR-1) to exert a protective effect and an anti-apoptosis effect [58,59]. Some studies found that APC induced EPCR- and PAR1-dependent anti-apoptotic signaling pathways in certain tumor cells that enhanced survival and increased the potential of these cells to form metastatic foci [60–62]. Furthermore, APC decreases apoptosis via p53 inhibition, the transcription factor that regulates pro-apoptosis gene expression [63].

Our recent study suggests that APC relieves acute ischemic injury in the heart through activating the AMPK signaling pathway and inhibition of NF-κB and JNK cascades which is independent of its anticoagulant function. In addition to that, APC can specifically increase the oxidation of glucose over fatty acids as substrates in the ischemia/reperfusion heart [64,65]. Therefore, APC has the potential protective effects against ischemia/reperfusion injury in the heart, however, the molecular mechanism stimulated by APC remains to be further investigated.

## A-769662

A-769662 is a small organic molecule that is a member of the thienopyrodine family of molecules. It was discovered as a direct activator of AMPK through screening of a chemical library of over 700,000 compounds [66]. A-769662 direct activation of AMPK is through binding which causes both inhibition of dephosphorylation of Thr172 and allosteric activation [67]. This is similar to the effect of AMP, however, the mechanism of AMPK activation by A-769662 is distinct from the mechanism of AMP and it is also specific to the β1 isoform of AMPK [68,69]. A-769662 has been shown to protect the heart from ischemia-reperfusion injury and exert cytotoxicity in PANC-1 pancreatic cancer cells via activation of AMPK [67,70]. There is evidence that A-769662 is a direct inhibitor of certain ATPase’s, including the Na+, K+-ATPase and some proteosomal ATPase’s [71]. These findings present the idea that A-769662 may also contribute, in addition to direct AMPK activation, to indirect AMPK inactivation through inhibiting the Na+, K+-ATPase and subsequently decreasing the AMP: ATP and ADP: ATP ratios in the cell [71]. The validity of this hypothesis remains to be tested as well as whether A-769662 is a general ATPase inhibitor, however, it will be important to determine any off-target effects of A-769662 in the activation of AMPK.

## Salicylate

Salicylates are one of the oldest used drugs by humans. Salicylate, originally derived from willow bark, has been widely replaced by its acetylated form, aspirin, for its medicinal effects. Aspirin is quickly broken down to salicylate once it enters circulation [72]. Salicylates are able to improve insulin sensitivity in obese mice and in humans affected by type 2 diabetes and they have also been shown to reduce the risk of several cancers including colon, breast, and prostate cancers [73–75]. It has recently been shown that salicylate directly activates AMPK in a mechanism similar to A-769662 and that aspirin was also able to activate AMPK in colorectal cancer cells [76,77]. However, it remains to be fully determined whether the improved diabetic and anticancer effects are directed through AMPK and also to what extent.

Aspirin is also used in treatment of cardiovascular disease in many cases, including during secondary prevention of vascular events in patients with history of cardiovascular disease, acute myocardial infarction, and, in some cases, primary prevention of vascular events [78]. The mechanism of this treatment is through the transfer of aspirin’s acetyl group, which is known to irreversibly inhibit the COX1 and COX2 enzymes and subsequently inhibit blood clotting caused by platelets [79]. However, activation of AMPK is implicated in pre-conditioning of the heart, and the activation of AMPK by salicylate may provide another mechanism in which aspirin is able to treat cardiovascular disease [80]. It has yet to be determined whether salicylate activates AMPK in the heart as well as the doses of aspirin needed to activate AMPK in the heart and if this will be low enough to avoid the detrimental gastrointestinal effects of aspirin.

It is interesting to note that salsalate, an orally available form of salicylate which does not inhibit clotting or cause detrimental gastrointestinal effects, is able to improve glucose homeostasis in patients with insulin resistance or type 2 diabetes [81–83]. Salsalate or other non-acetylated forms of aspirin may show greater promise in certain treatments of type 2 diabetes, cancer, and cardiovascular disease for their ability to be given at higher doses than aspirin. Some off-target effects of salicylate include its ability to uncouple mitochondrial respiration, which contributes further to AMPK activation, and inhibition of prostanoid biosynthesis and the protein kinase IB kinase β in the NF-κB pathway [76,84]. These pathways may also play an important part in the therapeutic effects of salicylate, so further research is needed to determine the specific role activation of AMPK plays in treatment of the previously mentioned diseases.

## MIF20

Macrophage Migrating Inhibitory Factor (MIF) is a cytokine expressed in several tissues including vascular smooth muscle and cardiomyocytes, and it is involved in controlling inflammation [85–87]. It was shown to stimulate AMPK in the ischemic heart, and, therefore, provide a possible target for upregulation of AMPK in treatment of cardiovascular disease [88]. The increased susceptibility of myocardial ischemia of older patients with history of cardiovascular disease may be attributed to an impaired MIF-AMPK activation response so upregulation of AMPK through targeting of MIF could provide a new treatment for these patients [89]. Recently, structure-based molecular designs of MIF antagonists led to the identification of a few MIF agonists [90]. One of these agonists, MIF20, was then shown to protect the heart from ischemic injury [91]. Because of the tissue-specific expression of MIF, a MIF agonist such as MIF20 may provide a unique way to up-regulate AMPK in the treatment of cardiovascular disease. It is interesting to note that in Non-Small Cell Lung Carcinomas (NSCLCs) the MIF pathway through CD74 has been shown to inhibit AMPK activation in contrast with its activation of AMPK in nonmalignant cell types [92].

## 5-Aminoimidazole-4-Carboxamide Ribonucleoside (AICAR)

AICAR is an analogue of adenosine that is metabolized into 5-aminoimidazole-4-carboxamide ribonucleoside monophosphate (ZMP) within cells [93]. Because of its similar structure to AMP, ZMP is able to directly activate AMPK through the same mechanism as AMP. This involves ZMP binding to the γ subunit of AMPK and promoting phosphorylation, inhibiting dephosphorylation, and allosterically activating AMPK as mentioned earlier, however, ZMP is not as effective an activator as AMP is [16,93]. AICAR has been shown to improve glucose homeostasis and improve insulin sensitivity in diabetic animal models, and low doses of AICAR and ionizing radiation in the human prostate cancer cell line PC3 can inhibit cell proliferation, decrease viability, increase apoptosis, and generate reactive oxygen species in a dose- and time-dependent manner [94]. *In vitro*, aged myofibroblast maturation can be rescued through AICAR activation of AMPK and its downstream kinase p38MAPK (a non-canonical TGF-β signaling pathway) [95]. However, AICAR has been mostly limited to use in studies to determine the downstream effects of AMPK activation because of limitations and adverse effects [96,97]. Some of the drawbacks of AICAR include that it is not orally available and must be intravenously injected [98]. Also, AICAR has variable potency, causes bradycardia, can lead to hypoglycemia, and it has deleterious effects on the blood lipid profile [96,98] (Table 1).

## Conclusions

AMPK plays a key role in the regulation of energy homeostasis within cells and at the whole-organism level both as a sensor and signaling molecule. Because of findings in the potential treatment of diseases such as type 2 diabetes, cancer, and cardiovascular disease with drugs that target and activate AMPK, it is important to identify and understand the mechanisms of the wide variety of drugs that activate AMPK. AMPK is widely expressed throughout the cell types of the body and is activated through both direct and indirect mechanisms, so it is also critical to take note of potential effects of these drugs beyond the cells of interest for treatment. It has already been shown that many of these drugs can have off-target effects.

Because there is tissue-specific expression of AMPK isoforms, another focus of research in the future may include tissue-specific targeting of AMPK activation. Direct activators of AMPK may provide the best possibility for this as they require specific binding to AMPK. It has already been shown that A-769662 and salicylate specifically target the β1 isoforms of AMPK, however, off-targets have already been identified for these drugs. Worth noting, a new small-molecule AMPK activator, ZLN024, has been shown to directly activate AMPK and have beneficial effects for db/db mice without affecting mitochondrial respiration or the ADP: ATP ratio of cells [99]. Findings such as this show the importance to keep identifying AMPK activators, as there may be more specific activators of AMPK with fewer off-targets possible that will lead to better treatment of diseases such as type 2 diabetes, cancer, and cardiovascular disease through AMPK activation.

## Figures and Tables

**Table 1:** AMPK activators and research stages in disease treatment.

Type of molecule	Diabetes	Cancer	Cardiovascular Disease
**Metformin**	Clinical Use [18]	Clinical Research [21] Cell Research [23]	Animal Research [32]
**Adiponectin**	Animal Research [40]	Cell Research [52]	Animal Research [51]
**APC**	Clinical Research [57]	Clinical Research [60–62] Cell Research [58,59]	Animal Research [63]
**A-769662**	Animal Research [66]	Cell Research [67,70]	Animal Research [67]
**Salicylate**	Clinical Research [81–83]	Cell Research [76,77]	Clinical Use [78]
**MIF20**	NA	Cell Research [92]	Animal Research [88]
**AICAR**	Animal Research [96,97]	Clinical Research [94]	Animal Research [95]
